# Analysis of Draize Eye Irritation Testing and its Prediction by Mining Publicly Available 2008–2014 REACH Data

**DOI:** 10.14573/altex.1510053

**Published:** 2016-02-11

**Authors:** Thomas Luechtefeld, Alexandra Maertens, Daniel P. Russo, Costanza Rovida, Hao Zhu, Thomas Hartung

**Affiliations:** 1 Center for Alternatives to Animal Testing (CAAT), Johns Hopkins Bloomberg School of Public Health, Environmental Health Sciences, Baltimore, MD, USA; 2 The Rutgers Center for Computational & Integrative Biology, Rutgers University at Camden, NJ, USA; 3 Department of Chemistry, Rutgers University at Camden, NJ, USA; 4 CAAT-Europe, University of Konstanz, Konstanz, Germany

**Keywords:** animal testing alternatives, ocular toxicity, *in silico*, dataset, chemical safety

## Abstract

Public data from ECHA online dossiers on 9,801 substances encompassing 326,749 experimental key studies and additional information on classification and labeling were made computable. Eye irritation hazard, for which the rabbit Draize eye test still represents the reference method, was analyzed. Dossiers contained 9,782 Draize eye studies on 3,420 unique substances, indicating frequent retesting of substances. This allowed assessment of the test’s reproducibility based on all substances tested more than once. There was a 10% chance of a non-irritant evaluation after a prior severe-irritant result according to UN GHS classification criteria. The most reproducible outcomes were the results negative (94% reproducible) and severe eye irritant (73% reproducible).

To evaluate whether other GHS categorizations predict eye irritation, we built a dataset of 5,629 substances (1,931 “irritant” and 3,698 “non-irritant”). The two best decision trees with up to three other GHS classifications resulted in balanced accuracies of 68% and 73%, i.e., in the rank order of the Draize rabbit eye test itself, but both use inhalation toxicity data (“May cause respiratory irritation”), which is not typically available.

Next, a dataset of 929 substances with at least one Draize study was mapped to PubChem to compute chemical similarity using 2D conformational fingerprints and Tanimoto similarity. Using a minimum similarity of 0.7 and simple classification by the closest chemical neighbor resulted in balanced accuracy from 73% over 737 substances to 100% at a threshold of 0.975 over 41 substances. This represents a strong support of read-across and (Q)SAR approaches in this area.

## 1 Introduction

In a parallel article (Luechtefeld et al., 2016, this issue), we describe the curation of the data made available to the public by the European Chemical Agency (ECHA) until mid-December 2014. ECHA chemical dossiers describe diverse chemical and toxicological studies. These dossiers contain studies mapping to over 300 EPA, OECD and EU guidelines. Automation of data extraction from ECHA dossiers enables analysis of testing redundancies, construction of computational models, evaluation of endpoint distributions and other data analyses. The status of ECHA and the REACH (Regulation (EC) No 1907/2006) legislation make the ECHA dossier database extremely valuable to computational toxicology, for the evaluation of many study protocols (*in vitro*, *in vivo*, read-across and QSAR methods), and for systematic analyses in general. In this article, we use this data to assess eye irritation testing.

Eye irritation is the production of changes in the eye following the application of a test substance to the anterior surface of the eye of rabbits, which are followed for reversibility for 21 days after application (OECD Test Guideline 405, *in vivo*) (OECD, 2012a), also known as the Draize rabbit eye test. Draize eye irritation presents one of the most criticized and contested animal tests still in use today. It has been the subject of criticism both on the basis of irreproducibility and subjectivity as well as animal welfare considerations, and its replacement has therefore been the target of alternative methods development (Wilson et al., 2015; York and Steiling, 1998). However, Draize testing has remained in use with only small modifications since 1944 (Draize et al., 1944).

Under the European chemicals legislation REACH, substances produced or imported in volumes greater than 1 ton per annum must be assessed for eye irritation potential. Substances belonging to the 1 to 10 ton per annum tonnage band should use *in vitro* methods; above this tonnage the use of the Draize test is recommended (Grindon et al., 2008). Recent progress in the validation of alternative methods (Vinardell and Mitjans, 2008; Hartung, 2010) supports their use in weight-of-evidence evaluations, but no method to fully replace the animal test has yet been accepted. Until now, three methods have been adopted by the Organization for Economic Cooperation and Development (OECD) as partial replacements of the Draize test to classify substances as inducing serious eye damage: These are two organotypic assays, the Bovine Corneal Opacity and Permeability (BCOP) test method (OECD test guideline (TG) 437) and the Isolated Chicken Eye (ICE) test method (OECD TG 438) (OECD, 2013a), both based on slaughterhouse materials, and a cell-based assay, the Fluorescein Leakage (FL) test method (OECD TG 460) (OECD, 2012b). Two of these alternative methods (BCOP and ICE) were recently adopted by the OECD also for the identification of substances not requiring a classification for serious eye damage/eye irritation (OECD, 2013a). Two other test methods, namely the cytosensor microphysiometer (Hartung et al., 2010) and the short-time exposure test (Sakaguchi et al., 2011; Takahashi et al., 2008), a cytotoxicity-based *in vitro* assay that is performed on a confluent monolayer of Statens Seruminstitut Rabbit Cornea (SIRC) cells, are currently in the process of regulatory acceptance by the OECD. Several other eye irritation methods are listed in the OECD test guideline proposals of 2015 (SkinEthic, *in vitro* macromolecular test, and others^1^). Finally, the EPA recently published strategies for testing antimicrobial cleaning products^2^.

The hope to develop testing strategies to replace the Draize test by combining several animal-free methods has raised expectations. Combination methods following the top-down bottom-up approach have been proposed (Scott et al., 2010; Kolle et al., 2011; Hartung, 2010).

The number of animals used for Draize testing is fairly small compared to the more demanding tests, e.g., for reproductive toxicity (Hartung and Rovida, 2009; Rovida and Hartung, 2009), which is owed to the small number of rabbits required per test article (i.e., 1–3 animals) according to a stepwise testing strategy in OECD Test Guideline 405 for the determination of the eye irritation/corrosion properties of substances. However, the severity of suffering and the limitations of the assay, noted as early as 1971 (Weil and Scala, 1971) and confirmed more recently (Adriaens et al., 2014), call for special attention.

The EU 7^th^ Amendment to the Cosmetic Directive (76/768/EEC), now Regulation 1223/2009, banned animal testing for new cosmetic ingredients and requires non-animal alternatives for safety assessment. These pressures motivate the creation of computational and *in vitro* test models for eye irritation tests and others (Hartung, 2008). However, the lack of large public databases of Draize results has inhibited the progress of computational modeling (Hartung and Hoffmann, 2009). Only most recently (Adriaens et al., 2014) a larger database was compiled from *in vivo* rabbit eye irritation data registered in the New Chemicals Database (NCD) of the former European Chemicals Bureau (ECB) and three reference substances databases (Eye Irritation Reference Substances Data Bank (ECE-TOC), the ZEBET database and the Laboratoire National de la Santé (LNS) database), which included, after a quality check of the Draize eye test data, 1,860 studies. However, this database is not publicly available.

Since the existing literature for eye irritation until recently lacked large reference datasets, QSAR and other *in silico* as well as integrated testing strategies were evaluated only for small datasets. In December 2014, Verma and Matthews described the evaluation of an FDA/CFSAN-developed artificial neural network for the prediction of eye irritation on 2,928 substances with specificities and sensitivities in the 80-90% range (Verma and Matthews, 2015). The construction of their database relied on manual curation of a large number of publications with Draize results (Cronin et al., 1994; Andersen, 1999; Bagley et al., 1999; Cho et al., 2012; Sugai et al. 1990, 1991). Their work shows the value of the increased size of a dataset, but their reliance on aggregation of literature results suffers from a lack of a central repository. We should not rely on literature aggregation for toxicological datasets if possible, as doing so is inherently error-prone and non-scalable to other endpoints.

This publication analyses results of Draize experiments and related data available in ECHA chemical dossiers^3^. We explore the internal reproducibility of Draize results in these dossiers and demonstrate simple models for the prediction of eye irritation using chemical structures, Globally Harmonised System (GHS) hazards and Draize endpoints (cornea, iris, conjunctivae and chemosis).

## 2 Methods

### 2.1 Database construction

The database for these analyses was created from ECHA dossier pages as described (Luechtefeld et al., 2016, this issue). Automated extraction by linguistic search engines of data from ECHA online dossiers enables analysis of diverse chemical study data. Extracted REACH data were stored as a queryable collection of documents in a Mongo database^4^ (Chodorow, 2013; Godbillon, 2015). Every document in the extracted database is identified by a unique set of three fields:

– *ECNumber:* substance identifier (e.g., “214-306-9”)
– *Type:* study description (e.g., “exp key acute toxicity dermal”)
– *Num:* disambiguates repeat studies (1, 2, 3,…)

Studies in ECHA contain fields for “materials and methods”, “results and discussions”, “administrative data” among others. The final extracted database contains over 10,000 dossiers representing a substantial but incomplete extraction of the entire ECHA repository. The resulting database on 9,801 substances encompasses 326,749 experimental key studies, additional dossier information on classification and labeling and other miscellaneous data. 3,420 substances contain studies for a Draize test and form the basis of this study.

### 2.2 Reproducibility assessment

We evaluate Draize reproducibility by answering, “What is the probability a Draize test outcome agrees with another Draize test outcome for the same chemical?” This question is answered by constructing conditional probabilities for each category:

$$P(T_{2}=1|T_{1}=1)=\frac{P(T_{2}=1\cap T_{1}=1)}{P(T_{1}=1)}$$

The above formula gives the probability of a Type 1 result for the Draize test given a Type 1 result for another Draize test of the same chemical. T_i_ = 1 represents a test (identified by the number i) with outcome Type 1. The given equation is simply the definition of conditional probability. This reproducibility refers to multiple tests in potentially different labs and should not be confused with traditional inter-/intralaboratory reproducibility.

### 2.3 Draize endpoint modeling

OECD TG 405, known as the Draize test, describes how data is obtained for scoring criteria for acute eye irritation/corrosion (OECD, 2012a). The Draize test involves application of a chemical of interest to white albino rabbit eyes *in vivo*. Damage is scored for cornea, iris, conjunctivae and chemosis. Each ocular endpoint has subjective scoring rules described in Table 1. GHS hazards describe how Draize endpoint scores can be mapped to 4 categories of irritation: Type 1, Type 2A, Type 2B and non-irritating. Type 1 and Type 2 irritants are differentiated by reversibility with Type 1 irritants causing serious or irreversible eye damage that persists for 21 days post-exposure. Type 2 irritants are reversible before 21 days. Type 2A and Type 2B irritants are differentiated by the severity of irritation with Type 2A irritants more severe than Type 2B and further subcategorized if effects are fully reversible within 7 days of substance application. Eye irritation categories are defined from endpoint features. The classification strategy for H318 (Draize Type 1), H319 (Draize Type 2A) and H320 (Draize Type 2B) is generated in Figure 4 from classifications given by the Infectious Disease Research Institute (IDRI)^5^ and defined by the UN GHS. OECD guideline data is interpreted according to the following rules (OECD, 2012a): Type 2A versus 2B can be determined by 7 day reversibility of effects. Severity of cornea and iris effects with 21-day reversibility differentiate Type 1 and 2A.

For each substance we derived from all the Draize studies an average value for each Draize endpoint (iris, cornea, etc.) and a maximum value for each endpoint. The ECHA Draize studies report Draize endpoint values, thus allowing for the sum and maximum values to be found for these endpoints.

In addition, we derived one “reversibility” feature matching the study and endpoint with the longest reversibility time. For example, for a chemical with a chemosis endpoint that shows a reversibility period greater than 21 days we apply the value “irreversible” to the “reversibility” feature. Finally, the classification and labeling hazard value reported in the given substance’s ECHA dossier was used to define a Draize GHS category corresponding to the category of Draize response (Type 1, 2A, 2B). The features for this model are described below:

1. *Chemosis mean:* chemosis mean scores
2. *Chemosis max:* max of chemosis scores for substance
3. *Iris mean:* mean iris scores
4. *Iris max:* max iris scores
5. *Cornea mean:* mean of cornea scores
6. *Cornea max:* max of cornea scores
7. *Conjunctivae mean:* mean of conjunctivae scores
8. *Conjunctivae max:* max of conjunctivae scores
9. *Reversibility:* longest endpoint reversal period
10. *Draize GHS Category:* H318 = Type 1, H319 = Type 2A, H320 = Type 2B

### 2.4 Decision tree construction

Decision trees constructed for prediction of eye irritation category from Draize endpoint features (iris, cornea, conjunctivae, reversibility, etc.) and Draize GHS categories (H318, H319, H320) used Weka’s J48 decision tree algorithm (Quinlan, 2014; Hall et al., 2009). Briefly, this algorithm works by iteratively selecting the attribute yielding the greatest reduction in entropy. Decision trees are useful for finding predictive rules and for visualizing relationships in the data.

### 2.5 K-nearest neighbor

Selection of PubChem fingerprints requires the mapping of EC-Numbers to PubChem chemical identifiers. The PubChem power user gateway was used for this purpose (Cheng et al., 2014). Similarity approaches require construction of chemical-chemical similarity and implementation of algorithms. PubChem 2D conformational substructure fingerprints were generated using the Chemistry Development Kit, an open-source Java chemistry package (Steinbeck et al., 2003). Weka’s IkB algorithm was used with k set to 1 and different thresholds selected for minimum similarity (Aha et al., 1991; Hall et al., 2009). PubChem 2D conformation chemical substructure fingerprints are binary vectors signifying the presence or absence of 881 different substructures. Chemical similarity approaches typically suffer from activity cliffs and poor accuracy when using small chemical datasets. We measured chemical similarity via the PubChem 2D conformational fingerprints and the Jaccard (Tanimoto) distance. This is a relatively simple approach to similarity; more advanced approaches include self-organizing maps, which could define similarity within the context of eye irritation categorizations.

The chemical similarity graph was constructed using the Fruchterman Reingold algorithm as implemented by Gephi with area = 1000, gravity = 10, speed = 1.0 (Fruchterman and Reingold, 1991; Bastian et al., 2009). This layout algorithm works via simulating a physical process whereby neighboring (similar) vertices attract each other and dissimilar vertices repel.

## 3 Results and discussion

**Fig. 1:**
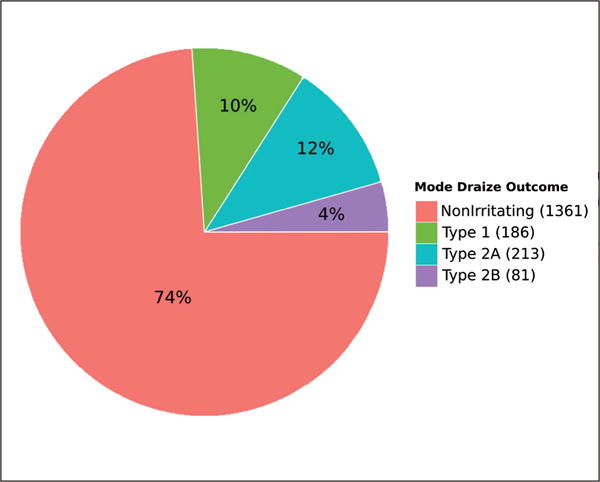
Prevalence of outcomes for substances tested with OECD TG 405 (Draize rabbit eye test) in REACH registrations 2008–2014 Mode outcome was used for substances with multiple OECD TG 405 studies.

**Fig. 2:**
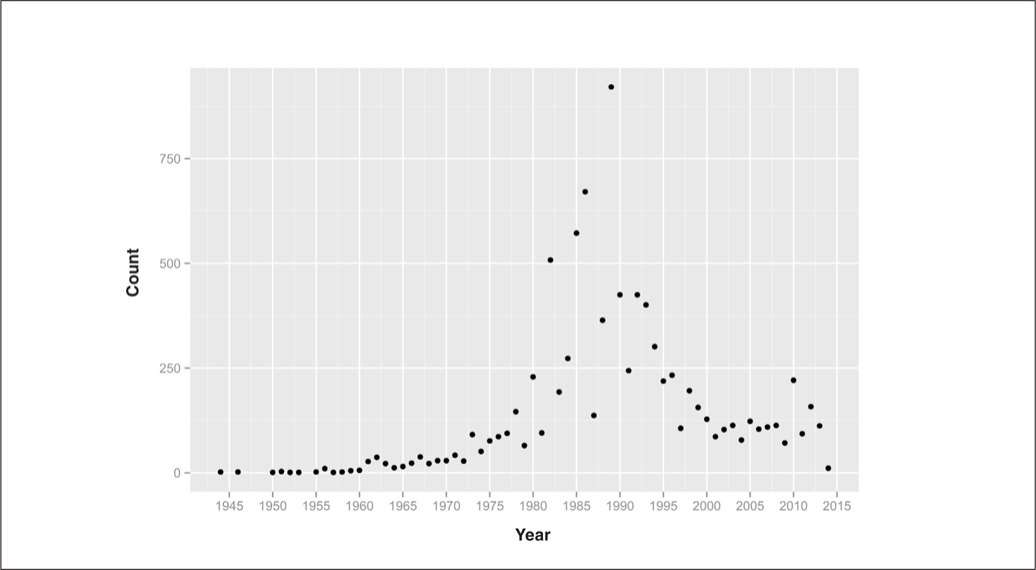
Number of Draize rabbit eye tests per year found in REACH registrations 2008–2014

In addition to allowing analysis via computational models, availability of large numbers of Draize studies allows for more generalized analyses. Many substances were tested in multiple Draize studies. Approximately 25% of the 1,841 substances for which a mode eye irritation category could be extracted are irritants. Figure 1 gives prevalence of the mode Draize outcome for each substance with at least one Draize study. Figure 2 shows the number of Draize studies per year (as defined by the ECHA reference date) and shows a rise and peak around 1985 with a decade long decline afterwards.

### 3.1 Analysis of Draize scoring

The mapping of Draize results to eye irritant categories is well defined. However, the scoring of individual endpoints is of varying degrees of subjectivity, and observations of reversibility may be more reproducible than observer assessment of damage (swelling, reddening, etc.) both in terms of inter-observer variation and animal variation. Therefore, one might expect eye irritant categories more dependent on subjective features to be less reproducible.

Investigation of acute eye toxicity reveals a large number of substances with relevant *in vitro* and repeated *in vivo* studies. In total 10,524 studies were extracted with the following characteristics:

1. “Eye” contained in study type
2. Materials and methods data exists
3. Results and discussions data exists
4. Klimisch reliability score of 1 or 2 (Klimisch et al., 1997), indicating reliability of the reporting of the data.

Of the 10,524 studies retrieved this way, 7,706 reported an *in vivo* OECD TG 405 (Draize test), 2,076 were read-across based on OECD TG 405 results from other substances, 292 report OECD TG 437 (Bovine Corneal Opacity Test) (OECD, 2013a), 41 report OECD TG 438 (Isolated Chicken Eye Test) (OECD, 2013b) and in 409 cases we were unsuccessful in extracting an associated OECD TG.

Surprisingly, out of 9,782 Draize studies (*in vivo* and read-across) there are only 3,420 unique substances, indicating frequent retesting of substances for eye irritation hazard (Tab. 2). Indeed, after skin irritation (OECD 404) and repeat dose toxicity (OECD 422), the Draize test is the most commonly executed OECD TG for a human health endpoint in the dataset.

**Fig. 3:**
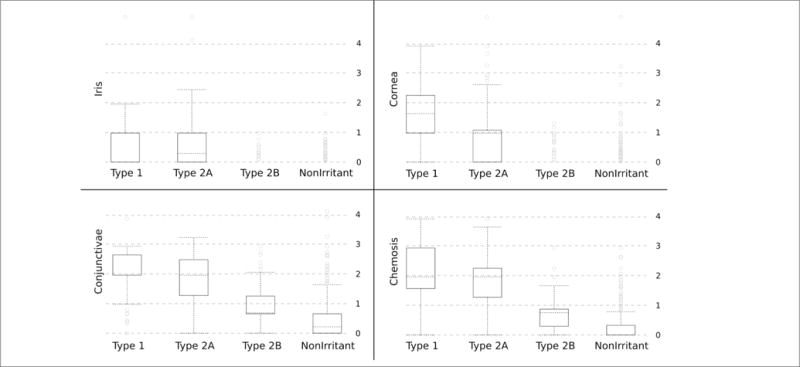
Eye irritation category endpoint scores Built from 4,134 Draize studies where the result could be mapped to a standard Draize category. Box plots describe score distributions for iris, cornea, conjunctivae and chemosis endpoints given different Draize categories and are based on the “score” parameter for Draize tests. Reversibility is not considered in this analysis. Scores outside of Draize definitions (given in Tab. 1) are the results of incorrect inputs in ECHA dossiers.

ECHA dossiers give eye irritation categorization in natural language with values such as “category 1”, “corrosive”, “cat. I”, “highly irritating”, etc. Test evaluation involves an irritation score for iris, conjunctivae, cornea and chemosis. Substances are categorized as Type 1 (“serious irreversible damage”), Type 2A (“reversible irritation”), Type 2B (“reversible mild irritation”) and non-irritating (Wilson et al., 2015). With knowledge of GHS criteria these study interpretations can be mapped to standard eye irritation categories through text analysis. Our approach to natural language text analysis could only map to the appropriate category with high confidence for 491 of the 1,279 substances with repeat studies. Figure 3 visualizes the relationship between irritation categories and scoring for iris, conjunctivae, cornea and chemosis, with more severe damage equating to a higher score (see Tab. 1).

Our analysis indicates a greater difference in observed severity between Type 2A and Type 2B than any other consecutive categories. This figure is built from 4,134 Draize studies, where the submitter’s interpretation could be mapped to a standard category. The difference, given by OECD, between Type 1 and Type 2 categories is a question of reversibility, whereas the difference between 2A and 2B is a question of severity (Wilson et al., 2015). The individual scores in Figure 3 describe severity irrespective of reversibility. While the subjective nature and subsequent variability of severity values is clear in Figure 3, we still see a strong reduction in severity scores in the progression from Type 1 to Type 2A, Type 2B and non-irritant categories.

By observing the dependency of eye irritation categories on severity data, we can speculate on the biological features of endpoint-specific data. The predication of Type 1/Type 2 on reversibility makes it unsurprising that Type 1/Type 2 categories are not well separated by severity scores. Conjunctivae scoring best separates categories and thus delivers the greatest information content. The cornea endpoint differentiates Type 1 and Type 2A more completely than other endpoints, suggesting that corneal damage repair is less probable than other endpoints. Chemosis and iris scoring show little separation between Type 1 and Type 2A, suggesting that these forms of damage are more easily repaired. The low prevalence of iris and cornea damage relative to conjunctivae and chemosis damage in the Type 2B category indicates that these endpoints are perhaps less sensitive to irritating substances. Alternatively, different Draize endpoints may be activated by different chemical/biological mechanisms.

With access to the specific results of the Draize studies, it would be possible to create models of each Draize endpoint, and perhaps thereby identify potential differential mechanisms of iris, cornea, conjunctivae or chemosis damage.

### 3.2 Reproducibility

In order to assess the reproducibility of Draize eye irritation scoring, conditional probabilities for each category were constructed: Table 3 considers the reported eye irritation categories for all substances with at least two Draize tests and an extractable eye irritation category (491 substances). For example, Table 3 gives a 10.4% chance of a non-irritant evaluation given a prior Type 1 evaluation. The most probable repeat test outcome given a result of Type 2A or Type 2B is non-irritant. The highest reliability values in Table 3 come from prior negative outcomes (94% probability of future negative outcome) and severe eye irritation (74% probability given same class prior).

When juxtaposed to Figure 3, the similarity between Type 2B and a non-irritant outcome becomes more apparent: 77 out of 86 substances with multiple Draize tests and at least one Type 2B result also have at least one result of non-irritant. In other words, it would appear that the Draize test cannot reliably distinguish between these categories – something that should be kept in mind when evaluating the reliability of an *in vitro* replacement or machine learning approach.

### 3.3 Modeling

Having established that Draize results are reproducible only with some caveats, we next attempted to build *in silico* models for the Draize eye test. We decided to model eye irritation category by using the follow features:

1. Features of the Draize test (endpoint mean, max and reversibility values see Section 2.3)
2. Other GHS hazards
3. Chemical similarity via substructure analysis.

By building a model based on features of the Draize test, we can determine whether the existing eye irritation classifications align with the rules given by GHS eye irritation hazard criteria. Modeling of Draize test results via other GHS hazards allows for consideration of redundant testing – if other GHS hazards have high positive or negative predictive value, then there is likely some potential for test reduction. Finally, analysis of Draize results via chemical substructures allows for visualization of the distribution of Draize types over the chemical universe. We should expect that sufficiently similar substances will have similar Draize outcomes. Cases where this hypothesis is not true may present opportunities to discover novel mechanisms of eye irritation or extend our understanding of the applicability of read-across.

#### 3.3.1 Draize endpoint modeling

Eye irritation categories were modeled from a number of endpoint features as described in Section 2.3. Substances for this dataset were filtered from all REACH substances by selecting only those with endpoint data for every feature and only using studies that match ECHA’s “exp key acute eye toxicity” label and OECD TG 405. The resulting dataset is composed of 391 substances (Tab. 4). A larger dataset of 1943 substances was also constructed by relaxing the “data required for every endpoint” requirement and achieved similar results (85% accuracy and a similar Classification And Regression Tree (CART)). Unfortunately only 6 Type 2B substances exist in this dataset, and this class value was discarded from the options due to underrepresentation.

**Fig. 4:**
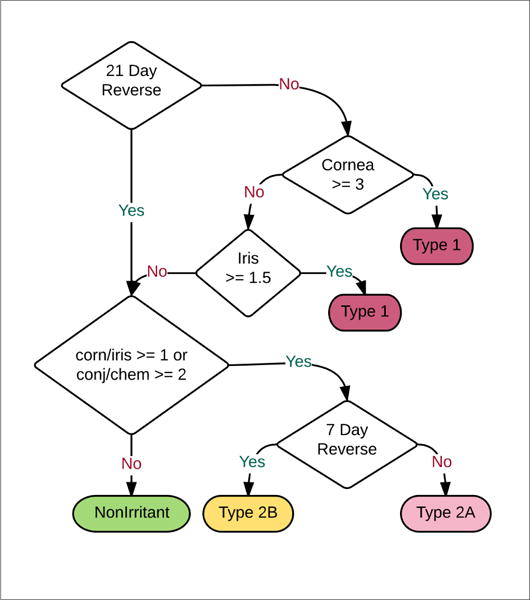
Draize endpoint classification strategy as represented by IDRI The flowchart shows how eye irritation classifications are made for Type 1, Type 2A, Type 2B and non-irritant categories. Corn = cornea score, chem = chemosis score, 7 Day Reverse = Status of 7 day phenotype reversibility, 21 Day reverse = Status of 21 day phenotype reversibility. It should be noted that substances causing serious or irreversible eye damage (“corrosion”) that persists within 21 days post-exposure are also considered Type 1.

These 9+1 features (see Section 2.3) and eye irritation category given for 391 substances are reduced into a classification and regression tree through the simple Quinlan approach of attribute selection via maximum information gain (Quinlan, 2014). An ideal decision tree should match closely with Figure 4, which is a human-made decision tree matching GHS criteria.

**Fig. 5:**
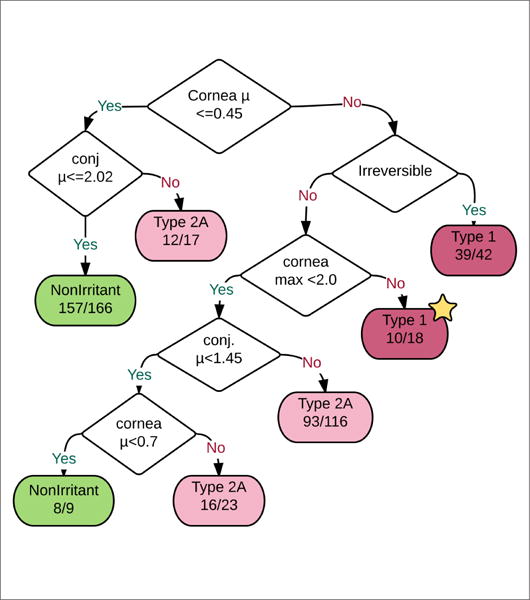
GHS Draize category decision tree from Draize endpoint data Decision tree trained using CART algorithm from severity and reversibility features using 391 substances for which eye irritation category could be defined. Note that this decision tree closely matches the criteria defined in GHS hazards. Cornea μ stands for the mean cornea score from Draize studies for a chemical, cornea max is the maximum observed cornea score from Draize studies for a chemical (see Section 2.3).

The decision tree resulting from the Quinlan approach (built from all data) is seen in Figure 5. This tree is in remarkable agreement with Figure 4. Differences in Figure 5 from Figure 4 are indicated by the yellow star for Type 1. Notably, only 10 of 18 substances falling into this errant leaf node held category Type 1. Cornea and conjunctivae thresholds identified by CART are close to those derived from GHS criteria, although the learned decision tree does fail to identify the difference between a corneal opacity score greater than or equal to 3.0 versus greater than or equal to 2.0.

The relatively strong reproduction of Figure 4 via decision tree learning and ECHA data indicates, as expected, that GHS acute eye hazard labeling is predictable algorithmically based on the Draize test outcomes and that our natural language based data extraction from ECHA is in good agreement with GHS values. It should be noted that individual animal data was not used in this analysis; only entire Draize tests and mean animal responses or maximum animal responses were applied.

#### 3.3.2 Modeling of Draize eye irritation outcomes from other GHS hazard classifications

Large datasets in ECHA dossiers can be used to identify testing redundancies and strategies. To evaluate redundancies within GHS categorizations, i.e., here whether eye irritation can be predicted from other hazards, we built a dataset of 5,629 substances classified as “irritant” if positive for H318, H319 or H320 and “non-irritant” otherwise. The resulting dataset contains 1,931 Draize irritants and 3698 non-irritants. The dataset contains “positive”, “negative”, or unknown values for 72 GHS hazards in the REACH extraction. Table 5 identifies individual GHS hazard-positive predictive values and hazard-negative predictive values. These are constructed for datasets consisting of all substances with a “positive” or “negative” value for Draize testing and the given hazard.

Hazards with less than 100 positive predictions (true positives + false positives) or less than 100 negative predictions (true negatives + false negatives) were filtered out. Notably, the physical hazards (H200s) and environmental hazards (H400s) are not very predictive of Draize outcome (with the exception of H290 “may be corrosive to metals”). Many of the health hazards are predictive. Of the health hazards (H300s), H302 (“harmful if swallowed”), H315 (“causes skin irritation”), H335 (“may cause respiratory irritation”) and H317 (“may cause allergic skin reaction”) all show high positive predictive values.

H312 “Harmful in contact with skin” has only an 80% positive predictive value for Draize hazards over 194 substances: this means 15 substances are positive for H312 and negative for H318, H319 and H320. Closer inspection of dossiers for three of these substances (ECNumbers 248-363-6, 200-858-8 and 248-363-6) reveal ECHA dossier evaluations of “conclusive but insufficient data for classification” for “serious eye damage/eye irritation”. Additionally, H318, H319 and H320 do not occur in the “hazard statements” field in these chemical dossiers. However, when these substances are inspected using the ECHA classification and labeling inventory database^6^, they are found to be positive for H318, H319 or H320 – indicating a disagreement between published ECHA dossiers and the C&L inventory for at least a subset of substances. Given these inconsistencies, we may expect other misclassifications of GHS hazards in the ECHA dossiers, which may explain other cases of lack of concordance (e.g., H314 “causes severe skin burns and eye damage” and Draize endpoints). Complete access to ECHA classification and labeling data would enable development of improved datasets for predicting eye irritation. The unintuitive nature of these hazard relationships makes human misclassification inevitable.

While these inconsistencies make modeling more difficult, we can still evaluate models by combining GHS values to predict H318, H319 or H320. To do this, we exhaustively searched all possible combinations of 3 hazards, for a total of 59,640 combinations (72!/69!*3!) and built datasets where the selected hazards had “positive” or “negative” values for each chemical in the subset. Decision trees were then built from these subsets with high-positive or high-negative predictive value.

**Fig. 6:**
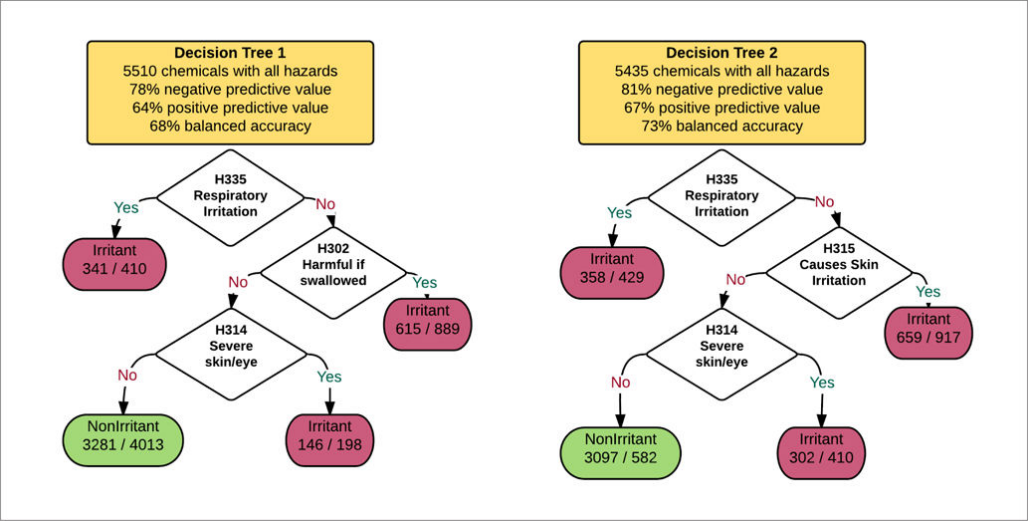
Decision trees built from subset analysis of hazards dataset Subsets generated from substances with hazard classifications for all hazards in decision tree. These decision trees indicate strong relationships between GHS hazard classifications.

The two presented subset trees (Fig. 6) were built by Weka software using the J48 algorithm corresponding to the rules “H335 or H302 or H314” and “H335 or H315 or H314”. These two decision trees resulted in balanced accuracies of 68% and 73%, which is within the range of accuracy of the Draize rabbit eye test itself. Noteworthy, both use inhalation toxicity data (“May cause respiratory irritation”), which are not typically available.

#### 3.3.3 Modeling of Draize eye irritation outcomes from chemical structure

**Fig. 7:**
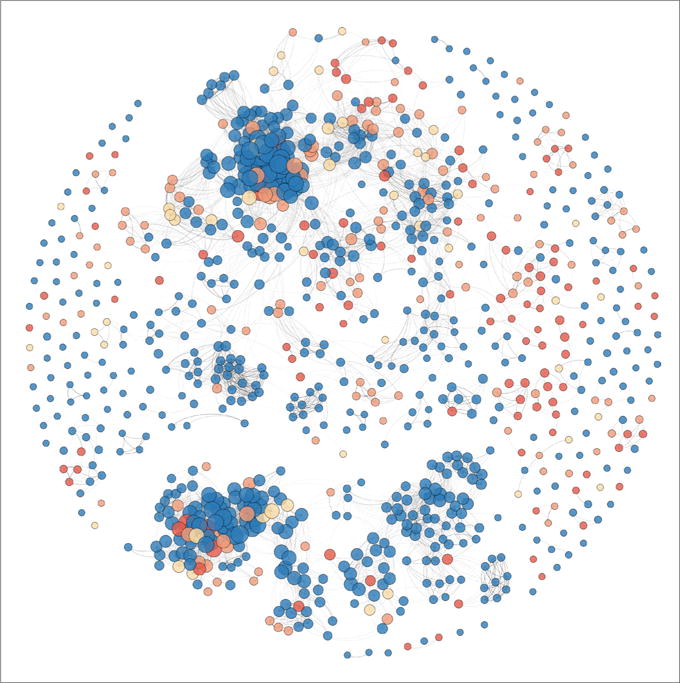
Fruchterman Reingold layout of a chemical similarity map for substances with rabbit eye irritation data in REACH registrations 2008–2014 929 substances with at least one Draize study and a mapping to PubChem were included. Chemical similarity was expressed as Jaccard (Tanimoto) index. Red = Type1, Orange = Type2A, Yellow = Type2B, Blue = non-irritant. Size of node is proportional to number of neighbors (larger nodes have more neighbors).

To evaluate the effectiveness of chemical structural similarity approaches for this hazard, we created a dataset of substances with at least one Draize study and a mapping to PubChem. The resulting dataset contains 929 substances, which can be found in PubChem. In Figure 7 we see a Fruchterman Reingold layout visualization of this similarity map. The map shows some clustering of Draize irritants (red, orange and yellow).

Next we tested the naïve approach to similarity modeling by using k-nearest neighbors with k set to 1. In this approach every chemical eye irritation category is predicted via the eye irritation category of the closest neighbor. We evaluated the models by setting different thresholds for the minimum allowed similarity. A chemical, *B*, is only used for prediction of another chemical, *A*, if it has similarity ≥ T, where T is a threshold. Table 6 shows the results of this analysis including the sensitivity, specificity and balanced accuracy for predicting chemical eye irritant/non-irritant. Starting with a threshold of 0.7, we see a resulting balanced accuracy of 73% over 737 substances. As the threshold is increased we see steady increases in balanced accuracy, sensitivity and specificity with a corresponding drop in the number of substances with at least one neighbor.

These strong balanced accuracies resulting from the simple approach of KNN with k = 1 and Tanimoto 2D structural distance lend credence to the similarity approach for chemical classification in the domain of Draize eye test classification. This represents a strong support of read-across and (Q)SAR approaches in this area, which can reduce testing with increasing confidence as larger datasets begin to cover more of the chemical universe.

In our search we found no existing satisfactory (Q)SAR models for eye irritation. However, the accuracies demonstrated here show promise for a potential similarity-based approach for eye irritation.

## 4 Conclusions

With 9,782 Draize studies on 3,420 unique substances, we created, based on ECHA’s publicly available registrations, a larger Draize dataset than any publicly available database. The fact that the ECHA database was not optimized for such data-mining creates some uncertainties as many text fields are not standardized, making queries difficult. A number of quality controls, consistency checks, and the plausibility of the overall results give this first analysis strong confidence. However, the demonstrated value of these data for the scientific community should urge a systematic publication of the REACH data.

The first assessment addresses the prevalence of this health hazard and its sub-categories: 34% of the substances were eye irritants, somewhat higher than suggested in an earlier analysis of the New Substances Database of the former ECB with 17.4% eye irritants (Adriaens et al., 2014) showing differences in the type of substances registered between 1981 and 2008 and those under REACH in the initial phase, i.e., predominantly high-production volume substances. This is important for the development of testing strategies (Hartung et al., 2013; Hoffmann et al., 2005; Rovida et al., 2015). The current analysis is certainly biased by the tiered introduction of substances into REACH. The first two deadlines included substances of higher tonnage levels and suspected carcinogenic, mutagenic and reproductive toxicants. However, a small number of new substances already has been introduced.

The extensive re-testing of substances documented here (up to 90 times for the two most commonly tested substances, 69 substances with 45 tests) allowed a thorough analysis of the reproducibility of the test. They confirm the reproducibility issues already described by Weil and Scala in 1971; very often this problem has been belittled by stating that these studies were done before OECD guideline standardization and GLP. They also confirm the assessments by Adriaens et al. (2014) about the test’s reproducibility. Their database includes fewer substances, but had access to the raw data, allowing intra-assay variability assessment. This demonstrates the extent to which access to the full REACH datasets could strengthen assessments. Analysis of the individual scores used to assign the overall eye irritation category showed some inconsistencies and redundancies, which could be useful, especially if the detailed information from the non-public parts of the dossiers is made accessible, for a possible revision of the scoring system.

The preliminary analysis and mining of the dataset shows that there is both considerable predictivity from chemical structure (our analysis based on the closest chemical neighbor with data) and biological activity (our analysis based on other GHS classifications). Neither alone has adequate accuracy to supplant the Draize test, although given the reproducibility problems of the assay, this result might actually be contested. Here, no attempt was made to use the information from chemico-physical properties, dedicated *in vitro* assays for eye irritation, toxicokinetic information or biological profiling as attempted in ToxCast^7^ or the Tox21^8^ program, all of which could likely considerably boost the predictive value of the knowledge-base. Follow-up research should focus on the integration of external databases with the ECHA data to create stronger models for eye irritation.

Making this dataset available will allow such analysis by the scientific community. The relatively impressive predictive value of the naïve approaches attempted here, however, strongly supports read-across (Patlewicz et al., 2014) and *in silico* approaches.

## Figures and Tables

**Tab. 1:** Description of Draize Scoring Rules

Endpoint	Description	Range
**Cornea**	degree opacity and ulcerations	0–4
**Iris**	swelling, hyperaemia	0–2
**Conjunctivae**	redness, vessel discernibility	0–3
**Chemosis**	swelling, lids closed/open	0–4

**Tab. 2:** Repetitions of TG 405 in REACH registrations 2008–2014

Repeats	Number of substances	Example ECNumber
90	2	613-683-0,295-445-2
45	69	940-595-2,295-431-6
18	1	931-203-0
15	2	700-762-0,692-840-5
13	38	934-268-3,931-515-7
12	2	918-317-6,500-513-4
11	2	931-700-2,226-109-5
10	2	232-395-2,939-581-9
9	1	267-291-6
8	27	940-730-5,940-728-4
7	32	940-727-9,940-726-3
6	75	931-745-8,300-226-2
5	56	939-578-2,939-575-6
4	135	939-693-8,939-621-5
3	254	939-715-6,939-688-0
2	593	208-778-5,941-224-7
1	2388	293-029-5,273-224-1

**Tab. 3:** Conditional probability of Draize evaluations given a previous test result Substances filtered to those with at least two Draize studies and extractable eye irritation category in REACH registrations 2008-2014 (491 substances).

Prior Type	1	2A	2B	Non	Total
**1**	73%	16.1%	0.4%	10.4%	46
**2A**	4.2%	32.9%	3.5%	59.4%	138
**2B**	0.2%	4%	15.5%	80.2%	86
**Non**	1.1%	3.5%	1.5%	93.9%	400

**Tab. 4:** Confusion Matrix for Dataset 1 CART

(row predicted, column class)	1	2A	Non
**1**	49	21	1
**2A**	7	121	9
**Non**	4	14	165

**Tab. 5:** All hazard classifications that make > 100 positive and negative predictions of Draize rabbit eye outcomes as given by hazard classifications for H318, H319 or H320 in REACH registrations 2008–2014

Hazard	PPV	TP+FP	NPV	TN+FN	BAC	Total	Description
H290	0.81	112	0.69	3627	0.53	3739	May be corrosive to metals
H301	0.60	200	0.67	5339	0.52	5539	Toxic if swallowed
H302	0.61	991	0.72	4550	0.61	554	Harmful if swallowed
H311	0.59	153	0.68	4682	0.52	4835	Toxic in contact with skin
H312	0.80	194	0.69	4640	0.54	4834	Harmful in contact with skin
H314	0.67	508	0.70	5030	0.57	5538	Causes severe skin burns and eye damage
H315	0.68	988	0.74	4553	0.64	5541	Causes skin irritation
H330	0.63	102	0.68	3306	0.52	3408	Fatal if inhaled
H331	0.64	176	0.69	3231	0.54	3407	Toxic if inhaled
H332	0.51	426	0.69	2987	0.55	3413	Harmful if inhaled
H334	0.61	122	0.69	2016	0.54	2138	May cause allergy or asthma symptoms…
H335	0.80	363	0.71	4069	0.59	4432	May cause respiratory irritation
H341	0.53	118	0.68	4857	0.51	4975	Suspected of causing genetic defects
H412	0.52	583	0.68	4677	0.54	5260	Harmful to aquatic life with long-lasting…

PPV = positive predictive value, TP+FP = true positives + false positives, NPV = negative predictive value, TN + FN = true negatives + false negatives, BAC = balanced accuracy. Total = the total number of substances that had both the given hazard and H318, H319, H320.

**Tab. 6:** K-nearest neighbor approach with K=1 for substances with rabbit eye irritation data in REACH registrations 2008–2014 929 substances with at least one Draize study and mapping to PubChem were included. Weka’s IkB algorithm was used with k set to 1 and different thresholds selected for minimum Jaccard (Tanimoto) similarity required for chemical prediction.

T	Substances	Sensitivity	TP+FN	Specificity	TN+FP	BAC
0.7	737	0.64	248	0.81	489	0.73
0.725	703	0.64	238	0.81	465	0.73
0.75	641	0.65	215	0.83	426	0.74
0.775	604	0.66	197	0.85	407	0.75
0.8	545	0.66	169	0.85	376	0.76
0.825	488	0.67	142	0.86	346	0.77
0.85	416	0.68	114	0.87	302	0.7
0.875	351	0.71	87	0.89	264	0.80
0.9	272	0.78	63	0.91	209	0.85
0.925	197	0.77	44	0.92	153	0.84
0.95	135	0.86	28	0.95	107	0.91
0.975	41	1.00	5	1.00	36	1.00

T = threshold, the minimum percent required to consider a chemical a neighbor. Substances = the number of substances with at least one neighbor meeting the threshold requirement. TP + FN = true positives + false negatives, TN + FP = true negatives + false positives, BAC = balanced accuracy.

## Footnotes

1 http://www.oecd.org/env/ehs/testing/TGP%20work%20plan_declassification_July%202015.pdf

2 http://www2.epa.gov/pesticide-registration/alternate-testing-framework-classification-eye-irritation-potential-epa

3 http://echa.europa.eu/

4 https://www.mongodb.org

5 http://www.idri.org/GHS/HazClassSummary.pdf

6 http://echa.europa.eu/information-on-chemicals/cl-inventory-database

7 http://www.epa.gov/comptox/toxcast/

8 http://www2.epa.gov/chemical-research/toxicology-testing-21st-century-tox21
